# What influences the clinical decision-making of dentists? A cross-sectional study

**DOI:** 10.1371/journal.pone.0233652

**Published:** 2020-06-05

**Authors:** Abdulrahman Ghoneim, Bonnie Yu, Herenia Lawrence, Michael Glogauer, Ketan Shankardass, Carlos Quiñonez

**Affiliations:** 1 Dental Public Health, Faculty of Dentistry, University of Toronto, Toronto, Ontario, Canada; 2 Periodontics, Faculty of Dentistry, University of Toronto, Toronto, Ontario, Canada; 3 Department of Health Sciences, Wilfrid Laurier University, Waterloo, Ontario, Canada; Navodaya Dental College and Hospital, INDIA

## Abstract

Clinical decision-making is a complex process influenced by clinical and non-clinical factors. The aim of this study was to investigate the association between provider, patient, and practice factors with clinical decision-making among dentists in Ontario, Canada’s most populated province and its largest dental care market. This was a cross-sectional, self-administered survey of a random sample of general dentists in Ontario (n = 3,201). The 46-item survey collected demographic, professional, and practice information. The outcome (treatment intensity) was measured using a set of clinical scenarios, which categorized dentists as either relatively aggressive or conservative in their treatment decisions. Associations were assessed using bivariate analysis and logistic regressions. One thousand and seventy-five dentists responded (33.6% response rate). Age (p = 0.001), place of initial training (p<0.001), number of dependents (p = 0.001), number of hygienists employed (p = 0.001), and perceptions of practice loans (p = 0.020) were associated with treatment intensity. Dentists who were <40-years old (OR = 2.06, 95% CI:1.39–3.06, p<0.001), American-trained (OR = 2.48, 95% CI:1.51–4.06, p<0.001), and perceived their practice loans as large (OR = 1.57, 95% CI:1.02–2.42, p = 0.039), were relatively more aggressive in their treatment decisions. Various non-clinical factors appear to influence the clinical decision-making of dentists in Ontario.

## Introduction

Clinical decision-making is foundational to the practice of dentistry. Some argue that a substantial portion of dental care lies in the “gray zones” where the defining criteria for a “right” or “wrong” treatment are unclear. [1] Regardless, due to the imbalance of knowledge between dentist and patient, the latter must rely on the former to provide information on the most appropriate procedure and clinical direction. [2, 3] As a result, a dentist is ethically obligated to provide the recommendation that best guards a patient’s well-being.

Many definitions of clinical decision-making exist, but in its simplest terms, it is the process of choosing between different alternatives or options. [4] Such decisions are rarely simple and involve a complex process that requires gathering and evaluating clinical and other information to formulate decisions. Hence, many factors influence clinical decisions. [5, 6] Research has explored some of these factors in medicine, nursing, and dentistry, and putative factors can be classified into “clinical” (or sometimes referred to as “technical”) and “non-clinical” factors. Clinical factors are described as the factors attributed to the patient’s health, such as their current disease status, symptoms of disease, and history and future risk of disease. Non-clinical factors are described as factors that influence the clinician’s behavior yet are not exclusively related to the patient’s clinical status, such as the patient’s race, socioeconomic status, health insurance status, as well as other personal characteristics pertaining to the clinician and patient. [7]

Among dentists, previous studies demonstrate associations between non-clinical factors such as provider age, years of experience and place of initial training with clinical decisions. [8] Grembowski et al. [9], for example, found that younger dentists tend to adopt more aggressive clinical approaches and perform more unnecessary treatments compared to their older peers. Gordon et al. [10] revealed that dentists from the United States are more prompt to intervene on carious enamel lesions, whereas dentists from Scandinavian countries (Denmark, Norway, and Sweden) would rather intervene when the carious lesion is into dentin. Zadik and Levin [11] found that unnecessary prescription of postoperative antibiotics and endodontic treatments are more prevalent among Latin American and Eastern European graduates, respectively. Grembowski et al. [12] and Gordan et al. [10] have shown that dentists in practices that are “not busy enough” tend to intervene significantly more often in the treatment of enamel surface lesions and tend to offer a higher numbers of services per patient after controlling for all other factors. The same association has been found between the type of practice (solo or small vs. large practices) and dentists’ decisions regarding restorative treatment. [13] To a lesser extent, factors such the reimbursement system in place or a patient’s insurance status have also been associated with dentists’ treatment decisions. [12, 14, 15]

Ultimately, while this evidence clarifies some of this dynamic, it is still limited in scope, as it has tended to only explore a small number of variables that might contribute to the decision-making of dentists, in addition to being limited by small sample sizes. Also, despite this evidence, the dynamics of what influences clinical decision-making is yet to be studied within a Canadian context. To this end, this study explores the association between provider, patient, and practice factors with clinical decision-making among a large and representative sample of general dentists in Ontario, Canada’s most populated and diverse province, and its largest dental care market.

## Materials and methods

This was a cross-sectional study conducted through a self-administered survey sent to a random sample of general dentists practicing in Ontario. The sampling frame (N = 7,067) was the 2016 register of the Royal College of Dental Surgeons of Ontario (RCDSO), the regulatory body for dentists in Ontario. The inclusion criterion was general practitioners in private practice with an RCDSO license, and the exclusion criteria were: 1) specialists; 2) those whose practice was not subject to the dental care market, such as public health dentists and university dental faculty members; and 3) those who participated in the pilot testing of the survey.

According to the sample size calculation proposed by Dillman, the required sample size was 1,067. [16] However, due to the traditional low response rate from dental professionals, this number was tripled, and 3,201 surveys were sent out. [17] The sample was selected using a random start systematic sampling technique.

**Fig 1:**
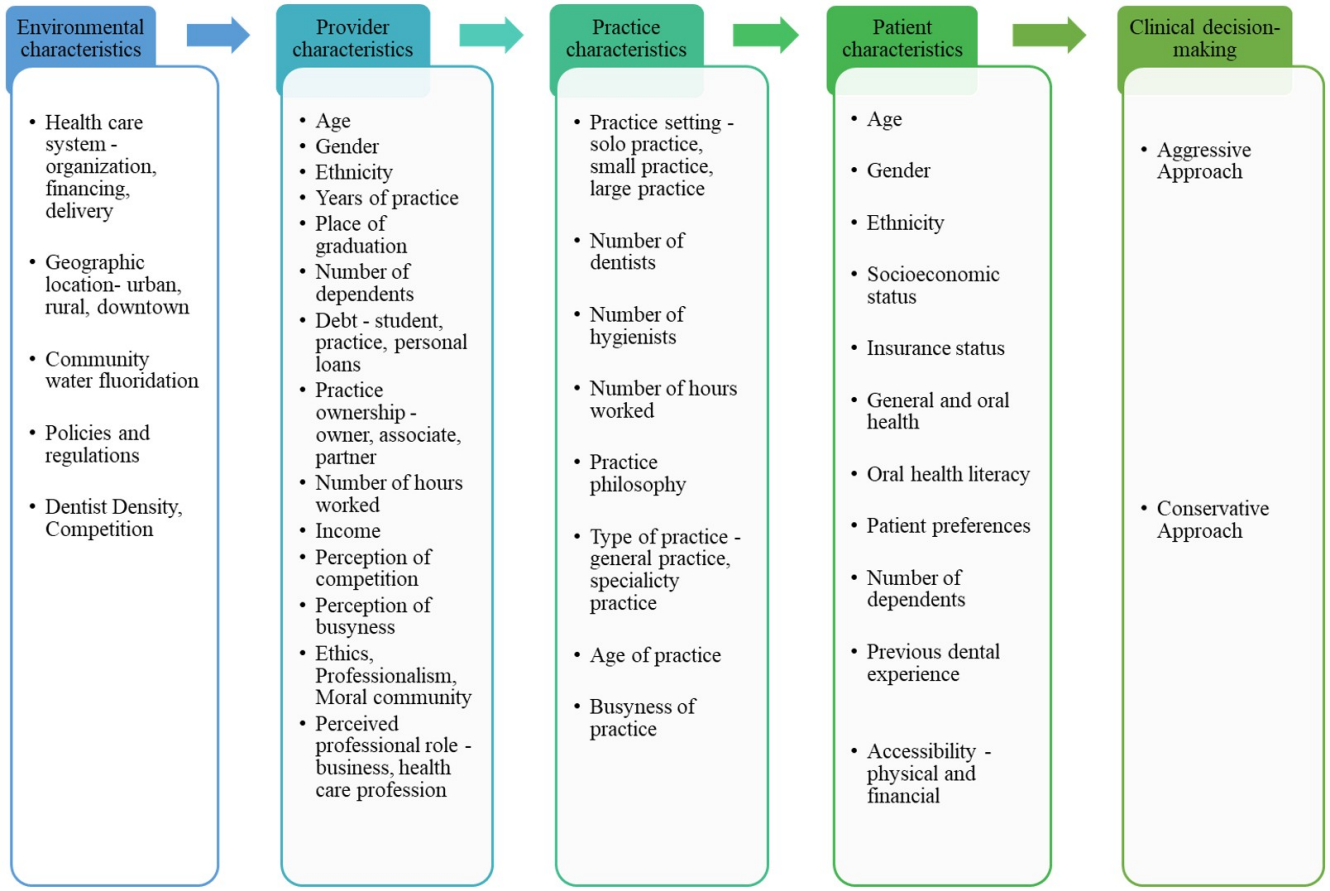
The conceptual framework of factors affecting dentists’ clinical decision-making.

After reviewing the health and dental care literature for factors associated with clinical decision-making, a conceptual framework for this study was developed (Fig 1). The framework was partly based on similar models established by Bader and Shugars [18] and Brennan and Spencer [19]. The framework putatively links dentists’ clinical decisions with factors grouped into environmental, practice, provider, and patient domains. Also, speculative factors where no empirical evidence was found were included into the conceptual framework based on anecdotal reports of their potential influence (e.g. amount required to bill/hour to be profitable, perception of other dentists, and the clinician’s number of dependents).

Using the framework, the 46-item survey was developed, which contained closed-ended questions to collect information on: a) provider characteristics (e.g. age, gender, place of initial training, number of dependents, perception of professional role and student loans); b) practice characteristics (e.g. age of practice, number of hygienists employed, percentage breakdown of routine work, satisfaction with practice busyness, and perception of practice loans); and c) patient characteristics (e.g. insurance status). Survey questions were either sourced from previous literature or developed completely de novo (e.g. treatment intensity score). The survey instrument and the case scenarios described below are presented in S1 Appendix.

To quantify the outcome clinical decision-making, a “treatment intensity score” was assigned using ten vignettes, which were developed based on common clinical situations. For each vignette, four treatment options were provided. The options spanned from a very conservative treatment approach, scored as ‘1’, to a very aggressive treatment approach, scored as ‘4’. Adding up the scores for the ten vignettes yielded a continuous score. This allows for a range of values with the minimum and maximum scores of 10 and 40, respectively. The higher the treatment intensity score, the relatively more aggressive the dentist’s treatment decisions were deemed to be. The case scenarios and categorizing answers were developed from the literature and with the help of expert advice from three content experts (two generalists and one specialist) at the University of Toronto’s dental faculty. Table 1 maps the survey questions to the domains outlined in the conceptual framework.

**Table 1:** Survey questions mapped to the conceptual framework.

Question number	Variable	Domain reflected in the conceptual framework
1	Gender	Provider characteristics
2	Age	
3	Place of initial training	
4	Year of graduation	
5	Number of years of practice	
6	Number of years of practice in Canada	
7	Case scenario	Clinical decision-making
8	Case scenario	
9	Case scenario	
10	Case scenario	
11	Number of hours worked/week	Provider characteristics
12	Amount billed/hour to profitable	Practice characteristics
13	Percentage of private insurance patients	Patient characteristics
	Percentage of public insurance patients	
	Percentage of out of pocket patients	
14	Number of dentists in clinic	Practice characteristics
15	Practice ownership	
16	Number of practices owned	
17	Time spent in each practice	
18	Perception of practice loans	
19	Practice age	
20	Number of hygienists	
21	Number of hygiene hours	
22	Case scenario	Clinical decision-making
23	Case scenario	
24	Case scenario	
25	Had student loans	Provider characteristics
26	Time taken to pay off student loans	
27	Perception of student loans	
28	Case scenario	Clinical decision-making
29	Case scenario	
30	Case scenario	
31	Number of patients seen/day	Practice characteristics
32	Personal gross billing	
33	Satisfaction with practice busyness	
34	Percentage of diagnostic and preventive services	
	Percentage of treatment services	
	Percentage of elective services	
35	Technologies used in practice	
36	Referral behaviours	
37	Subjects of continuing education	Provider characteristics
38	Perceived professional role	
39	Perception of other dentists	
40	Perception of pressure from other dental clinics	Provider characteristics
41	Moral community	
42	Moral community	
43	Moral community	
44	Primary income earner	
45	Number of dependents	
46	Annual after-tax income	

Importantly, the results from three case scenarios showed very little variability in the responses. Subsequently, statistical analysis was completed excluding these three scenarios. When these three case scenarios were removed, the minimum (and most conservative) score became ‘7’ and the maximum (and most aggressive) score became ‘28.’ Also, score proportioning was performed for participants who did not complete the full set of questions. Proportioning was performed using the following formula, $\frac{Score\;based\;on\;the\;completed\;questions\;}{Number\;of\;questions\;answered\;}\times 7\times 100$. For instance, if a participant completed five questions with a score of 16, then their adjusted score is $\frac{16}{5}\times 7\times 100\;=\;22.4≅22$. Only the scores of respondents who answered five clinical scenarios or more were included in the data analysis. This resulted in the exclusion of the treatment scores of twenty-one respondents (2.0% of the sample size).

The survey was piloted with twenty general dentists for face validity and ease of completion, and any proposed modifications were discussed with the main research team (AG, BY, CQ) and undertaken as needed. Approval for the study (protocol number 00033950) was obtained from the Health Sciences Research Ethics Board at the University of Toronto in February 2017. Participation in the study was completely voluntary and all participants were informed about the purpose of the study and consented by filling in and returning the questionnaire.

In terms of data analysis, we underwent two types of statistical tests. First, we conducted our analysis based on a dichotomized outcome. The treatment intensity score was dichotomized with the median score used as the cut-off point. Respondents who scored less than the median were categorized as relatively conservative, while those who scored at or above the median were categorized as relatively aggressive. We performed bivariate tests (chi-square) on all the independent variables to explore if differences were associated with the outcome (relative aggressiveness vs. relative conservative). Independent variables with multiple levels were tested to the dependent variable at once. The level of significance for the bivariate analysis was set at p<0.1. Then, the significant variables from the bivariate analysis were carried forward to perform binary logistic regression. Finally, the significant variables from the binary logistic regression were then entered into a multivariable logistic regression using the block method adjusting for all variables simultaneously. This was done to facilitate data analysis and presentation and to identify the factors that differentiate dentists who are conservative and aggressive in treatment decisions in relative terms. In other words, the goal was to assess dentists’ clinical leanings rather than make normative statements about their clinical decisions.

Alternatively, simple, and multiple linear regressions were carried out to test the relationship of each exploratory variables with the treatment intensity score as a continuous variable. This was particularly useful in observing the changes in the treatment intensity scores per unit change in the predictor variables. Variables significant at the p<0.1 level in the simple linear regression model were included in the multiple linear regression analysis. All statistical analyses were performed using SPSS v.23. Finally, we created a correlation matrix to assess for collinearity between the independent variables. S2 Appendix outlines the matrix.

## Results

After excluding returned surveys for reasons such as that the dentist had moved or retired or that the survey was filled out twice by the same respondent, the study had 1,075 usable surveys (33.6% response rate). Tables 2 and 3 present the descriptive characteristics of the sample. To assess the representativeness of the sample, the demographic characteristics of respondents were compared to the members of the Ontario Dental Association (ODA), a voluntary professional association representing over 90% of Ontario dentists. [20] The respondents of the survey were comparable to the ODA’s records in terms of gender and place of initial training but not for age or year of graduation, with the sample overrepresented by older dentists. S3 Appendix outlines the full comparison.

**Table 2:** Descriptive characteristics (categorical variables)^1^.

Variable	n (% of total)
Socio-demographics	
**Gender**	1070
Male	701 (65.5)
Female	369 (34.5)
**Age**	1069
40 and younger	154 (14.4)
41 to 50 years	274 (25.6)
51 to 60 years	325 (30.4)
61 and older	316 (29.6)
**Place of initial training**	1070
Canadian dental school	807 (75.4)
American dental school	84 (7.9)
International dental school	179 (16.7)
**Year of graduation**	1033
Before 1980	220 (21.3)
1980–1989	302 (29.2)
1990–1999	296 (28.7)
2000–2009	160 (15.5)
2010–2016	55 (5.3)
**Total years of practice**	1068
0–10 years	106 (9.1)
More than 10 years	962 (90.9)
**Years of practice in Canada among those that were internationally or American-trained**	252
0–10 years	56 (22.2)
More than 10 years	196 (77.8)
**Primary income earner**	1047
No	122 (11.7)
My partner and I contribute equally	177 (16.9)
Yes	748 (71.4)
**Number of dependents**	1066
0	158 (14.8)
1	233 (21.9)
2–4	610 (57.2)
5 or more	65 (6.1)
**Annual personal after-tax income**	902
Less than $100,000	203 (22.5)
$100,000–150,000	267 (29.6)
$150,000–200,000	170 (18.8)
$200,000–250,000	98 (10.9)
$250,000 or more	164 (18.2)
Clinical characteristics	
**Number of hours worked/week**	1059
Less than 20 hours	121 (11.4)
20–35 hours	474 (44.8)
35–50 hours	433 (40.9)
More than 50 hours	31 (2.9)
**Number of dentists in practice**	1059
1	372 (35.1)
2–4	606 (57.2)
5 or more	81 (7.6)
**Practice ownership**	1061
Associate	283 (26.7)
Owner/Partner	778 (73.3)
**Number of practices owned/partnered in**	771
1	677 (87.8)
2 or more	94 (12.2)
**Practice age**	771
0–10 years	109 (14.1)
More than 10 years	662 (85.9)
**Number of hygienists employed**	772
0	82 (8.0)
1	128 (16.6)
2	196 (25.4)
3	158 (20.5)
4	121 (15.7)
5 or more	107 (13.9)
**Number of hygiene hours/week**	709
Less than 20 hours	61 (8.6)
20–35 hours	167 (23.6)
35–50 hours	175 (24.7)
More than 50 hours	306 (43.2)
**Number of patients seen/day**	1068
1–9 patients	569 (53.3)
More than 9 patients	499 (46.7)
**Personal gross billing income/day**	1038
Less than $1500	169 (16.3)
$1500–2000	155 (14.9)
$2000–2500	210 (20.2)
$2500–3000	183 (17.6)
$3000–3500	115 (11.1)
$3500 or more	206 (19.8)
**Percentage of patients with private insurance**	975
0–69%	400 (41.0)
70–100%	575 (59.0)
**Percentage of patients with public insurance**	977
0–9%	409 (41.9)
10–100%	568 (58.1)
**Percentage of patients paying out of pocket (OOP)**	977
0–19%	484 (49.5)
20–100%	493 (50.5)
**Percentage of preventive procedures**	1031
0–15%	514 (49.9)
16–100%	517 (50.1)
**Percentage of treatment procedures**	1029
0–59%	496 (48.2)
60–100%	533 (51.8)
**Percentage of elective procedures**	1031
0–19%	480 (46.6)
20–100%	551 (53.4)
**Number of technologies used**	1061
0	122 (11.5)
1	414 (39.0)
2	273 (25.7)
3	143 (13.8)
4 or more	106 (10.0)
Perceptions	
**Perceived professional role**	926
Health care professional	852 (92.0)
Business person	74 (8.0)
**Perception of other dentists**	917
Colleague	751 (81.9)
Competitor	166 (18.1)
**Had student loans**	1072
Yes	524 (48.9)
No	548 (51.1)
**Time taken to pay student loans**	512
Less than 1 year	84 (16.4)
1–5 years	255 (49.8)
5–10 years	90 (17.6)
More than 10 years	28 (5.5)
My student loans are not yet paid off	55 (10.7)
**Status of student loan**	512
Student loans paid off	457 (89.3)
Student loans not paid off yet	55 (10.7)
**Perception of student loans**	517
Small	194 (37.5)
Medium	175 (33.8)
Large	148 (28.6)
**Satisfaction with practice busyness**	999
Very satisfied	289 (28.9)
Somewhat satisfied	459 (45.9)
Somewhat dissatisfied	206 (20.6)
Very dissatisfied	45 (4.5)
**Perception of practice loans**	767
No practice loans	335 (43.7)
Small	165 (21.5)
Medium	150 (19.6)
Large	117 (15.3)
**Perception of pressure from other dental clinics**	1077
No pressure	333 (31.5)
Small	365 (34.5)
Medium	237 (22.4)
Large	122 (11.5)

This table was previously published in Ghoneim A, Yu B, Lawrence HP, Glogauer M, Shankardass K, Quiñonez C. Does competition affect the clinical decision-making of dentists? A geospatial analysis. Community Dent Oral Epidemiol. 2019;00:1–11. https://doi.org/10.1111/cdoe.12514

**Table 3:** Descriptive characteristics (continuous variables).

	Percentage of private insurance	Percentage of public insurance	Percentage of out-of-pocket	Percentage of diagnostic and preventive procedures per week	Percentage of treatment procedures per week	Percentage of elective procedures per week
N valid	977	977	977	1034	1034	1034
Missing	98	98	98	43	43	43
Mean	65.7	15.2	19.1	20.2	57.5	22.3
Median	70.0	10.0	20.0	15.9	60.0	20.0
Mode	70.0	10.0	20.0	10.0	70.0	10.0
Standard Deviation (SD)	20.8	17.9	12.2	15.5	18.2	16.0
Minimum	0	0	0	0	0	0
Maximum	100	96	100	95	100	100
Percentiles						
25^th^	60.0	5.0	10.0	10.0	45.0	10.0
75^th^	80.00	20.00	25.00	29.4	70.0	30.0

In terms of the primary outcome, the distribution of treatment intensity scores ranged from a minimum score of ‘7’ to a maximum score of ‘25’. The mean and mode of the distribution were 14.7 and 15.0, respectively. The reported 50^th^ and 90^th^ percentiles were 15.0 and 19.8, respectively. This indicates that, overall, dentists tended to report relatively conservative treatment approaches.

Table 4 presents the binary and multivariable logistic regression findings. In the binary logistic regression, a positive correlation can be observed between age and treatment intensity. Dentists who belong to the youngest age group, 40 years and younger, have 100% greater odds (OR: 2.06; 95% CI: 1.39–3.06, p<0.001) of reporting relatively aggressive treatment decisions than those 61 years and older. A similar trend is observed with year of graduation. Regarding place of initial training, graduates from American dental schools have 150% greater odds (OR: 2.48; 95% CI: 1.51–4.06, p<0.001) of reporting relatively aggressive treatment decisions than those who graduated from Canadian schools. Practice ownership also demonstrated an association; dentists who owned or were a partner in their practices had 30% greater odds (OR: 1.33; 95% CI: 1.01–1.75, p = 0.232) of reporting aggressive treatment decisions than dentists who were associates. However, this is not statistically significant. Further, dentists who perceive themselves as business people have 60% greater odds (OR: 1.59; 95% CI: 0.98–2.58, p = 0.063) of reporting relatively aggressive treatment decisions than those who perceived themselves as healthcare professionals.

**Table 4:** Binary and binomial logistic regression models presenting the odds of adopting relatively aggressive treatment decisions^2^.

	Model 1 Unadjusted Odds ratio* (95% CI)	P	Model 2 Adjusted Odds ratio† (95% CI)	P
Socio-demographic				
Gender				
Male (reference)	1.00	-	-	-
Female	1.26 (0.98, 1.63)	0.075	-	-
Years of practice (continuous)	0.98 (0.96, 0.99)	<0.001	-	-
Age				
40 and younger	2.06 (1.40, 3.06)	<0.001	-	-
41–50	1.69 (1.21, 2.34)	0.002	-	-
51–60	1.40 (1.02, 1.92)	0.036	-	-
61 and older (reference)	1.00	-	-	-
Year of graduation				
Before 1980 (reference)	1.00	-	1.00	-
1980–1989	1.44 (1.01, 2.06)	0.046	1.83 (1.05, 3.21)	0.034
1990–1999	1.89 (1.32, 2.71)	<0.001	1.77 (0.97, 3.22)	0.061
2000–2009	2.03 (1.34, 3.09)	0.001	1.76 (0.89, 3.50)	0.105
2010–2016	3.21 (1.71, 5.60)	<0.001	3.08 (1.30, 7.29)	0.011
Years of practice categorized				
Less than 10 years	1.45 (0.96, 2.19)	0.075	-	-
More than 10 years (reference)	1.00	-	-	-
Place of initial dental training				
Canadian dental school (reference)	1.00	-	1.00	-
American dental school	2.48 (1.52, 4.06)	<0.001	2.97 (1.36, 6.48)	0.006
International dental school	0.76 (0.55, 1.06)	0.108	0.69 (0.42, 1.14)	0.151
Number of dependents				
0 (reference)	1.00	-	1.00	-
1	0.63 (0.42, 0.95)	0.029	1.03 (0.55, 1.93)	0.925
2–4	1.16 (0.82, 1.66)	0.401	1.35 (0.77, 2.37)	0.295
5 or more	0.88 (0.49, 1.57)	0.654	0.76 (0.30, 1.90)	0.553
Annual personal after-tax income				
Less than 100,000 (reference)	1.00	-	1.00	-
100,000–150,000	1.30 (0.90, 1.89)	0.167	1.160 (0.676, 1.992)	0.590
150,000–200,000	1.72 (1.14, 2.61)	0.011	1.63 (0.87, 3.05)	0.130
200,000–250,000	1.65 (1.01, 2.69)	0.044	1.35 (0.64, 2.87)	0.432
More than 250,0000	1.80 (1.18, 2.74)	0.006	1.40 (0.69, 2.85)	0.355
Clinical characteristics				
Number of hours worked/week				
Less than 20 hours (reference)	1.00	-	1.00	-
20–35 hours	1.80 (1.19, 2.71)	0.005	0.65 (0.33, 1.28)	0.213
35–50 hours	1.53 (1.01, 2.32)	0.043	0.57 (0.28, 1.18)	0.131
More than 50 hours	1.75 (0.78, 3.92)	0.173	0.41 (0.11, 1.49)	0.177
Amount to bill per hour per chair to be profitable				
Less than $200 (reference)	1.00	-	1.00	-
200–300	1.96 (1.26, 3.05)	0.003	1.26 (0.69, 2.31)	0.460
300–400	1.73 (1.11, 2.71)	0.017	1.20 (0.62, 2.31)	0.582
400–500	2.66 (1.60, 4.42)	<0.001	1.77 (0.82, 3.83)	0.147
More than 500	1.89 (1.18, 3.02)	0.008	2.25 (0.97, 5.21)	0.059
Percentage of private insurance				
0–69% (reference)	1.00	-	1.00	-
70–100%	1.38 (1.07, 1.79)	0.014	1.20 (0.81, 1.75)	0.331
Percentage of public insurance				
0–9% (reference)	1.00		-	-
10–100%	0.80 (0.62, 1.04)	0.092	-	-
Practice ownership				
Associate (reference)	1.00	-	1.00	-
Partner/Owner	1.33 (1.01, 1.75)	0.044	1.25 (0.78, 2.00)	0.357
Number of practices dentist is owner/partner				
1 (reference)	1.00		-	-
2 or more	1.31 (0.843, 2.028)	0.232	-	-
Number of hygienists				
0 (reference)	1.00	-	-	-
1	3.15 (1.61, 6.15)	0.001	-	-
2	2.90 (1.54, 5.48)	0.001	-	-
3	4.13 (2.14, 7.95)	<0.001	-	-
4	3.32 (1.69, 6.52)	<0.001	-	-
5	3.69 (1.85, 7.35)	<0.001	-	-
Number of patients/day				
0–9	1.31 (1.03, 1.67)	0.030	1.61 (1.07, 2.43)	0.023
9 or more (reference)	1.00	-	1.00	-
Personal gross billing/day				
Less than $1500 (reference)	1.00	-	1.00	-
1500–2000	1.42 (0.91, 2.22)	0.120	1.25 (0.64, 2.45)	0.517
2000–2500	1.60 (1.06, 2.42)	0.026	1.48 (0.75, 2.93)	0.260
2500–3000	1.76 (1.15, 2.70)	0.010	1.90 (0.90, 3.98)	0.091
3000–3500	1.59 (0.98, 2.59)	0.059	2.09 (0.87, 4.85)	0.099
3500 or more	2.09 (1.40, 3.14)	<0.001	2.10 (0.92, 4.81)	0.079
Percentage of diagnostic and preventive procedures/ week				
0–15% (reference)	1.00	-	1.00	-
16–100%	0.71 (0.55, 0.91)	0.006	0.79 (0.54, 1.15)	0.209
Perceptions				
Perception of practice loans				
No outstanding loans (reference)	1.00	-	-	-
Small	1.34 (0.92, 1.96)	0.126		
Medium	1.76 (1.18, 2.61)	0.005	-	-
Large	1.57 (1.02, 2.42)	0.039		
Status of student loans				
Loans paid off (reference)	1.00		-	-
Loans not paid off yet	2.75 (1.47, 5.14)	0.002	-	-
Perception of student loans				
Small (reference)	1.00		-	-
Medium	1.26 (0.83, 1.90)	0.282	-	-
Large	1.48 (0.96, 2.29)	0.078	-	-
Satisfaction with practice busyness				
Very satisfied (reference)	1.00	-	1.00	-
Satisfied	1.33 (0.98, 1.79)	0.065	1.45 (0.93, 2.28)	0.104
Dissatisfied	1.58 (1.10, 2.27)	0.013	2.38 (1.32, 4.30)	0.004
Very dissatisfied	1.33 (0.70, 2.51)	0.382	3.17 (1.14, 8.78)	0.027
Perceived dentist role				
Healthcare professional (reference)	1.00	-	-	-
Business person	1.59	0.063	-	-
Perception of other dentists				
Strongly a colleague (reference)	1.00	-	1.00	-
Colleague	1.40 (1.02, 1.94)	0.040	1.53 (0.98, 2.39)	0.061
Competitor	1.05 (0.69, 1.59)	0.812	1.21 (0.67, 2.19)	0.538
Strongly a competitor	1.77 (1.01, 3.10)	0.046	2.23 (0.94, 5.27)	0.068
Perceived pressure from other dental clinics				
No pressure/small amount (reference)	1.00	-	1.00	-
Medium/Large pressure	1.32 (1.02, 1.70)	0.037	0.90 (0.57, 1.52)	0.658

* Model 1 entered all the variables independently

^†^ Model 2 entered significant variables (p<0.05) from Model 1, adjusting for all variables simultaneously.

Variables “perception of practice loans”, “status of student loans”, and the “number of hygienists employed” were not included in Model 2 as they only pertain to practice owners.

Variables “age” is highly correlated with “years of practice” and “year of graduation” (Spearman’s correlation -0.910 and 0.936 respectively), hence, only “year of graduation” was included in Model 2.

This table was previously published in Ghoneim A, Yu B, Lawrence HP, Glogauer M, Shankardass K, Quiñonez C. Does competition affect the clinical decision-making of dentists? A geospatial analysis. Community Dent Oral Epidemiol. 2019;00:1–11. https://doi.org/10.1111/cdoe.12514

Among dentists who have student loans, those who perceive these loans to be large have 50% greater odds (OR: 1.48; 95% CI: 0.96–2.29, p = 0.078) of reporting relatively aggressive treatment decisions than those who perceive their loans as small. Similarly, dentists who perceive their practice loans as large or medium have 60% (OR: 1.57; 95% CI: 1.02–2.42, p = 0.039) and 80% (OR: 1.76; 95% CI: 1.18–2.61, p = 0.005) higher odds of reporting relatively aggressive treatment decisions, respectively, than those with no loans.

Other variables such as the number of dependents, the amount billed per hour to be profitable, and the number of hygienists employed demonstrate significant associations with odds of adopting aggressive treatment behaviours. However, the relation does not seem to be changing ordinally for every level above the reference group (i.e. non-linear relationship).

In the multivariable regression, year of graduation, place of initial training, satisfaction with practice busyness, and perception of other dentists demonstrated the strongest associations with reporting aggressive treatment decisions after adjusting for all other variables simultaneously.

As per the linear regression, similar observations were noted. That being, younger dentists, those who graduated from American dental schools, those who had not paid off their student loans, that perceived their practice loans to be medium or large, perceived themselves as business people, were dissatisfied with their practice busyness, and perceived the competitive pressures from other dental clinics to be large reported higher treatment intensity scores. After controlling for all other variables, the multiple linear regression analysis showed that treatment intensity scores was significantly associated with the place of initial training (American dental school β: unstandardized partial regression coefficient = 0.84, international dental school β = -0.70), the number of patients seen/day (9 or more patients/day β = -0.80), gross billing income/hour (3500 or more gross billing/hour β = 0.85), the amount billed/hour to be profitable ($200-300/hour β = 0.98, $300-400/hour β = 1.01, $400-500/hour β = 1.35, $500 or more β = 1.86), perceived professional role (business person β = 0.83), and perceived pressure from other dental clinics (medium pressure β = 0.75). Tables 5 and 6 outline the findings from the simple and multiple linear regressions, respectively.

**Table 5:** Simple linear regression of the treatment intensity scores represented as a continuous variable.

Variable	Unstandardized coefficient	Standard Error	p-value	95% CI lower bound	95% CI upper bound
Age					
40 and younger	1.45	0.35	0.000	0.77	2.14
41 to 50 years	1.05	0.30	0.000	0.47	1.63
51 to 60 years	0.38	0.28	0.175	-0.17	0.94
61 years and older (constant)	14.10	0.20	0.000	13.71	14.49
Gender					
Male (constant)	14.53	0.14	0.000	14.26	14.80
Female	0.47	0.23	0.044	0.01	0.92
Place of initial training					
Canadian dental school (constant)	14.68	0.13	0.000	14.43	14.93
American dental school	1.14	0.409	0.005	0.34	1.94
International dental school	-0.49	0.30	0.099	-1.07	0.09
Year of graduation					
Before 1980 (constant)	13.92	0.22	0.000	13.49	14.36
1980–1989	0.58	0.30	0.055	-0.01	1.18
1989–1999	1.08	0.31	0.000	0.48	1.68
2000–2009	1.38	0.36	0.000	0.68	2.08
2010–2016	1.89	0.53	0.000	0.85	2.92
Years of practice					
Less than 10 years (constant)	15.21	0.34	0.000	14.54	15.88
10 years or more	-0.59	0.36	0.106	-1.29	0.12
Years of practice in Canada					
Less than 10 years (constant)	15.21	0.34	0.000	14.54	15.88
10 years or more	-0.59	0.36	0.106	-1.29	0.12
Primary income earner					
No (constant)	14.76	0.30	0.000	14.18	15.34
My partner and myself contribute equally	-0.12	0.40	0.775	-0.90	0.67
Yes	-0.07	0.32	0.831	-0.70	0.57
Number of dependents					
0 (constant)	14.58	0.28	0.000	14.04	15.13
1	-0.65	0.37	0.078	-1.36	0.07
2–4	0.43	0.31	0.173	-0.19	1.05
5 or more	0.06	0.53	0.914	-0.98	1.09
Annual after-tax income					
Less than 100k (constant)	14.41	0.19	0.000	14.04	14.77
100-150k	0.09	0.29	0.768	-0.48	0.65
150-200k	0.82	0.33	0.014	0.17	1.48
200-250k	0.42	0.41	0.301	-0.38	1.22
More than 250k	0.61	0.34	0.070	-0.05	1.28
Number of hours worked/week					
Less than 20 hours (constant)	14.02	0.307	0.000	13.42	14.62
20–35 hours	0.87	0.349	0.018	0.14	1.51
35–50 hours	0.69	0.353	0.049	1.00	1.39
More than 50 hours	0.92	0.722	0.201	-0.49	2.34
Number of dentists					
1 dentist (constant)	14.48	0.18	0.000	14.12	14.84
2–4 dentists	0.25	0.24	0.284	-0.21	0.71
5 or more dentists	0.87	0.44	0.047	0.01	1.73
Practice ownership					
Associate (constant)	14.36	0.21	0.000	13.95	14.77
Owner/partner	0.46	0.25	0.064	-0.03	0.94
Number of practices					
1 practice (constant)	14.61	0.12	0.000	14.38	14.84
2 or more practices	0.91	0.39	0.019	0.15	1.68
Practice age					
Less than 10 years	0.53	0.36	0.149	-0.19	1.24
10 years or more (constant)	14.64	0.12	0.000	14.41	14.87
Number of hygiene hours					
Less than 20 hours (constant)	14.18	0.17	0.000	13.84	14.52
20–35 hours	0.52	0.33	0.110	-0.12	1.16
35–50 hours	0.93	0.33	0.005	0.29	1.56
50 hours or more	0.99	0.27	0.000	0.46	1.52
Number of patients seen/day					
1–9 patients (constant)	14.86	0.15	0.000	14.57	15.16
More than 9 patients	-0.37	0.22	0.093	-0.81	0.06
Gross billing/day					
Less than $1500 (constant)	13.78	0.25	0.000	13.29	14.28
$1500–2000	0.64	0.38	0.096	-0.11	1.39
$2000–2500	0.97	0.35	0.006	0.28	1.66
$2500–3000	1.20	0.37	0.001	0.48	1.92
$3000–3500	1.11	0.42	0.008	0.29	1.94
$35000 or more	1.56	0.36	0.000	0.86	2.25
Amount to bill/hour to be profitable					
Less than $200 (constant)	13.41	0.25	0.000	12.91	13.91
$200–300	1.24	0.32	0.000	0.61	1.87
$300–400	1.41	0.33	0.000	0.76	2.06
$400–500	1.76	0.39	0.000	0.99	2.52
$500 or more	2.51	0.40	0.000	1.72	3.30
Perceived professional role					
Healthcare professional (constant)	14.62	0.11	0.000	14.40	14.85
Business person	0.95	0.43	0.027	0.11	1.80
Perception of other dentists					
Colleague (constant)	14.64	0.12	0.000	14.41	14.88
Competitor	0.31	0.31	0.302	-0.28	0.91
Had student loans					
Yes	0.17	0.221	0.43	-0.26	0.61
No (constant)	14.61	0.154	0.000	14.31	14.91
Time taken to payoff student loans					
Less than 1 year (constant)	14.66	0.14	0.000	14.40	14.93
1 to 5 years	-0.02	0.26	0.938	-0.54	0.50
5 to 10 years	0.11	0.41	0.797	-0.70	0.92
10 years or more	0.88	0.70	0.213	-0.50	2.26
Status of student loans					
Student loans paid off (constant)	14.61	0.11	0.000	14.39	14.83
Student loans not paid off yet	1.62	0.50	0.001	0.63	2.61
Perception of student loans					
Small (constant)	14.56	0.13	0.000	14.30	14.82
Medium	0.29	0.30	0.344	-0.31	0.88
Large	0.63	0.33	0.053	-0.01	1.27
Satisfaction with practice busyness					
Very satisfied (constant)	14.17	0.19	.000	13.79	14.55
Satisfied	0.61	0.26	0.018	0.11	1.112
Dissatisfied	0.45	0.49	0.361	-0.51	1.41
Very dissatisfied	1.22	0.32	0.000	0.60	1.84
Perception of practice loans					
No loans (constant)	14.30	0.14	0.000	14.02	14.58
Small loans	0.68	0.31	0.030	0.07	1.29
Medium loans	1.17	0.33	0.000	0.54	1.81
Large loans	1.13	0.36	0.002	0.42	1.84
Perception of pressure from other dental clinics					
No pressure (constant)	14.21	0.19	0.000	13.83	14.58
Small pressure	0.43	0.27	0.111	-0.10	0.96
Medium pressure	0.93	0.30	0.002	0.33	1.52
Large pressure	1.16	0.38	0.002	0.41	1.90

**Table 6:** Multiple linear regression.

Variable	Unstandardized coefficient	Standard Error	p-value	95% CI lower bound	95% CI upper bound	Variance Inflation Factor (VIF)
Age: 61 years and older as a reference						
40 and younger	0.56	0.64	0.384	-0.70	1.82	4.626
41 to 50	0.37	0.40	0.348	-0.41	1.15	2.689
Gender: Male as a reference						
Female	0.43	0.25	0.094	-0.07	0.93	1.314
Place of initial training: Canadian dental school as a reference						
American dental school	0.84	0.41	0.041	0.04	1.64	1.104
International dental school	-0.70	0.32	0.027	-1.33	-0.08	1.266
Year of graduation: 1980 and before as a reference						
1981 to 1990	0.38	0.31	0.221	-0.23	1.00	1.777
1991–2000	0.07	0.41	0.873	-0.74	0.87	3.033
2001–2010	-0.09	0.61	0.877	-1.30	1.11	4.321
2010–2016	0.09	0.84	0.912	-1.55	1.73	3.099
Number of dependents: No dependents as a reference						
1 dependent	-0.52	0.28	0.057	-1.06	0.02	1.147
Annual after-tax income: Less than $100,000 as a reference						
150-200k income	0.46	0.30	0.119	-0.119	1.045	1.055
Number of hours worked/week: Less than 20 hours worked as a reference						
20–35 hours worked	0.19	0.34	0.570	-0.47	0.86	2.541
35–50 hours worked	-0.06	0.36	0.869	-0.77	0.65	2.829
Number of dentists in the clinic: One dentist as a reference						
5 or more dentists	0.59	0.41	0.153	-0.22	1.39	1.071
Practice ownership: Associate as a reference						
Owner/partner	-0.43	0.34	0.207	-1.09	0.24	2.083
Number of practices owned/partnered in: One practice as a reference						
Own two or more practices	0.44	0.40	0.273	-0.34	1.21	1.136
Number of hygiene service hours/week: Less than 20 hours/week as a reference						
35–50 hygiene hours	0.43	0.34	0.212	-0.25	1.16	1.426
50 or more hygiene hours	0.49	0.31	0.116	-0.12	1.09	1.755
Number of patients seen/day: Less than 9 patients/day as a reference						
9 or more patients seen/day	-0.80	0.24	0.001	-1.27	-0.33	1.288
Gross billing income/hour: Less than $1500/hour as a reference						
1500–2000 gross billing/hour	0.23	0.39	0.546	-0.52	0.99	1.665
2000–2500 gross billing/hour	0.55	0.37	0.139	-0.18	1.27	1.936
2500–3000 gross billing/hour	0.72	0.40	0.072	-0.07	1.50	2.03
3000–3500 gross billing/hour	0.58	0.46	0.204	-0.32	1.48	1.79
3500 or more gross billing/hour	0.85	0.43	0.050	0.00	1.69	2.596
Amount billed/hour to be profitable: Less than $200/hour as a reference						
200–300 amount billed/hour to be profitable	0.98	0.32	0.002	0.35	1.61	1.958
300–400 amount billed/hour to be profitable	1.01	0.34	0.003	0.34	1.68	2.029
400–500 amount billed/hour to be profitable	1.35	0.41	0.001	0.55	2.14	1.720
500 or more amount billed/hour to be profitable	1.86	0.44	0.000	1.00	2.72	1.827
Perceived professional role: Healthcare provider as a reference						
Business person	0.83	0.42	0.049	0.00	1.66	1.044
Current status of student loans: Student loans are paid off as a reference						
Student loans not paid	0.92	0.59	0.121	-0.24	2.08	1.500
Perception of student loans: Small student loans as a reference						
Large student loans	-0.36	0.37	0.334	-1.09	0.37	1.464
Satisfaction with practice busyness: Very satisfied as a reference						
Satisfied	0.25	0.24	0.304	-0.23	0.72	1.291
Very dissatisfied	0.77	0.32	0.016	0.14	1.39	1.429
Perceived pressure from practice loans: No outstanding loans as a reference						
Small pressure from practice loans	0.46	0.33	0.168	-0.20	1.12	1.314
Medium pressure from practice loans	0.75	0.35	0.036	0.05	1.44	1.354
Large pressure from practice loans	0.40	0.40	0.320	-0.39	1.20	1.418
Perceived pressure from other dentists: No perceived pressure as a reference						
Medium pressure from other dentists	0.35	0.27	0.201	-0.18	0.88	1.137
Large pressure from other dentists	0.55	0.36	0.135	-0.17	1.26	1.214

## Discussion

The results of this study suggest an association between various non-clinical factors and dentists’ clinical decision-making in a representative sample of dentists in Ontario, Canada’s most populated and diverse province, and its largest dental care market. The findings are corroborated in the existing literature. For example, previous studies have reported that older dentists make more conservative treatment decisions. [8, 12, 21, 22] It might be that the experience accumulated over years of practice allows dentists to be a better judge of clinical cases. [22] Others believe that older dentists are more ethically inclined and less pressured by financial incentives when recommending procedures. [23]

Place of initial training has also been found to be associated with differences in dentists’ treatment decisions. [11, 24, 25] One hypothesis that can explain these differences is the variation in dental curricula and clinical practices taught in different international settings. It has been suggested that such differences would fade away as time practiced in the host country increases, as practitioners adapt to the oral health needs and professional culture of the respective population. [25] However, in this study, the years practiced in Canada was not significantly associated with clinical decision-making.

Perception of practice loans and perception of practice busyness were also significantly associated with clinical decision-making in this study. It might be that dentists who are less busy and perceive their loans to be large tend to recommend more involved and higher-cost treatments. Previous studies have found a similar association between practice busyness and treatment decisions. [9, 22] Financial challenges facing dentists, such as outstanding educational loans and the perception of large practice loans, were all pointing in the same direction, too. Despite the absence of published empirical evidence to support these findings, anecdotally, it is suggested that when facing financial hardships, dentists may overtreat or recommend unnecessary procedures to alleviate some of their financial pressures. [26]

A fundamental argument that has presented itself in dentistry is whether dentists are health care professionals and/or business persons. [27] Dentistry in general is described as a profession, which assumes that the professional “professes” to protect and foster “the benefit of the public”. [28] This means that the patient’s welfare is always prioritized over those of the practitioner’s. [28] However, some argue that the values and norms of dentistry, as a health professional culture, often conflict with the demands of its other culture, namely that of business, which emphasizes profit and high income as priorities. [29] This can manifest when dentists prioritize those who demand costly interventions (veneers) over those who are in more need of less costlier procedures (simple restorations) to maximize profit. This could explain the differences in clinical decision-making between those who consider themselves primarily health care professionals compared to business persons.

There have been previous attempts to quantify clinical decision-making among dentists. The most popular method appears to be through assessing the depth of a carious lesion at which a dentist would restoratively intervene based on radiographic images. [8, 13, 30] Another method includes “ethical” calibration of recommended treatment options to a hypothetical vignette, [31] and comparing differences between treatments proposed and delivered by dentists under different reimbursement systems. [14, 32] Importantly though, to our knowledge, our study is the first to use an aggregated treatment intensity score utilizing common clinical situations.

The most significant shortcoming of this study is the potential presence of social desirability bias when answering the vignettes and other questions. Respondents may tend to provide answers based on textbook recommendations, which might not necessarily mirror their actual clinical practices. This bias may also persist despite the confidentiality promised to participants. This represents one of the reasons for dichotomizing the outcome, aside from the simplicity of data analysis and presentation. The analysis tried to identify the factors associated with dentists’ leanings in clinical decision-making (relatively conservative vs. relatively aggressive). While there are consequences to dichotomizing a continuous variable, such as the loss of information or the misclassification of respondents, the authors believe that, in this data set, where the responses are not normally distributed, dichotomization offered a simpler and potentially more valid way of representing the outcome.

Another limitation of this study is the underrepresentation of younger dentists (14% compared to 29% in the ODA’s membership) within the sample. This has arguably led to the underestimation of the effects of age, which was significantly associated with the primary outcome. This has relevance to our decision not to employ weighting adjustment due to the unavailability of demographic and other descriptive data about Ontario dentists. We recognize this as a shortcoming that hinders the generalizability of our findings. Also, it is important to consider the study design when interpreting results, thus due to its cross-sectional nature, causation cannot be inferred. For instance, based on the findings from this study, we cannot tell if those who have fewer patients choose to do more aggressive, time filling procedures, or those who like to do more aggressive procedures have fewer patients because there is no time to squeeze others in. Finally, while we acknowledge the inevitable presence of moderate data collinearity (VIF scores between 1–5), between some of our independent variables, we believe that it does not impact on the validity of our findings in a significant way, hence not warranting the need for corrective measures.

The strengths of this study include the achievement of the minimum sample size, which allows for, within its limits, the generalization of the findings to the entire population of practicing dentists in Ontario, as the data was collected province-wide by utilizing a comprehensive sampling frame (i.e. all registered dentists in Ontario). In addition, this study was robust compared to previous studies exploring similar outcomes, as it investigated more than thirty variables potentially associated with clinical decision-making. Further, from a methodological standpoint, the study presents a potentially innovative method to quantify clinical decision-making and presents an opportunity for formal exploration of its reliability and validity through future research. Despite the contribution this study provides to the clinical decision literature in dentistry, the data suggest that we have a long way to go before we fully understand what impacts dentists’ treatment decisions. Future research should investigate the impact of other important environmental and patient characteristics on dentists’ clinical decisioning such as regulations on dental advertising, patient insurance coverage, and patient demand dental procedures.

The results of this study have numerous educational and professional implications. Some of the educational implications include the potential need to train students to deal with the anticipated financial stresses of clinical life and emphasizing ethical principles in practice. From a professional standpoint, it is important that the public perceives dental professionals as their health advocates, first and foremost. Yet, unfortunately, due to the arguably prevalent shift in the mindset of dental practices towards a business model, the erosion of public trust is a serious consequence facing the profession. [33] Strengthening the ethical reasoning of dentists can arguably mitigate financially driven treatment decisions, which in return can mitigate the general undermining of public trust.

## Conclusion

The results from this study suggest an association between non-clinical factors and dentists’ self-reported treatment decisions. This is the first study to explore the factors potentially contributing to the clinical decision-making of dentists in Canada. Moreover, it serves as a foundation for further studies exploring factors thought to influence dentists’ treatment decisions using a novel measurement approach.

## Supporting information

S1 Appendix The survey instrument. (PDF)

S2 Appendix The correlation matrix. (PDF)

S3 Appendix Demographic comparison. (PDF)
