# Correction: What influences the clinical decision-making of dentists? A cross-sectional study

**DOI:** 10.1371/journal.pone.0253183

**Published:** 2021-06-08

**Authors:** Abdulrahman Ghoneim, Bonnie Yu, Herenia Lawrence, Michael Glogauer, Ketan Shankardass, Carlos Quiñonez

The Competing Interests statement is incorrect. The correct Competing Interests statement is as follows: The authors have read the journal’s policy and have the following competing interests: CQ receives consulting income for dental care related issues from Green Shield Canada. This does not alter our adherence to PLOS ONE policies on sharing data and materials. There are no patents, products in development or marketed products associated with this research to declare.
