# Supplementary material for: What influences the clinical decision-making of dentists? A cross-sectional study
Source: PLoS One. 2020 Jun 5;15(6):e0233652. doi: 10.1371/journal.pone.0233652 (PMC7274387; doi:10.1371/journal.pone.0233652)
Supplement: S1 Appendix — (PDF) [file pone.0233652.s001.pdf]

Appendix 1. The survey instrument

This survey will consist of a mixture of questions about yourself, your dental office(s), and your practice of clinical dentistry via case scenarios.

First, some questions about yourself.

1. You are?

- ☐ Male
- ☐ Female

2. What is your age?

- ☐ Less than 31
- ☐ 31 to 40
- ☐ 41 to 50
- ☐ 51 to 60
- ☐ 61 and older

3. Where did you receive your initial dental training?

- ☐ Canadian dental school
- ☐ American dental school
- ☐ International dental school

4. What year did you graduate from your initial dental training? __________

5. Considering all jurisdictions, how many years have you been in practice?

- ☐ 0-5 years
- ☐ 6-10 years
- ☐ More than 10

6. If you are American or internationally trained, how many years have you been practicing in Canada?

- ☐ 0-5 years
- ☐ 6-10 years
- ☐ More than 10

Now some case scenarios.

Please read the following case scenarios and select the answer that is closest to what you would do for each patient.

7. A 20-year-old male patient presents to your clinic for a regular check-up. Today's radiographs and clinical examination show he has impacted 18 and 28 and fully erupted 38 and 48. He mentions he has had slight discomfort on 38 in the past. How would you proceed?

- ☐ Advise on the importance of extracting third molars because they will be harder to extract as he gets older.
- ☐ Take a "wait-and-see" approach.
- ☐ Extract 38, and refer him to an oral surgeon to extract 18, 28.
- ☐ Extract 38, 48 and monitor 18, 28.

8. The images below refer to a 25-year-old female patient with a history of depression and poor oral hygiene. She presents to your clinic complaining of dry mouth. At what caries lesion depth do you think it would be best to do a permanent restoration instead of trying a preventive therapy? Please circle one answer.

1 2 3 4 5

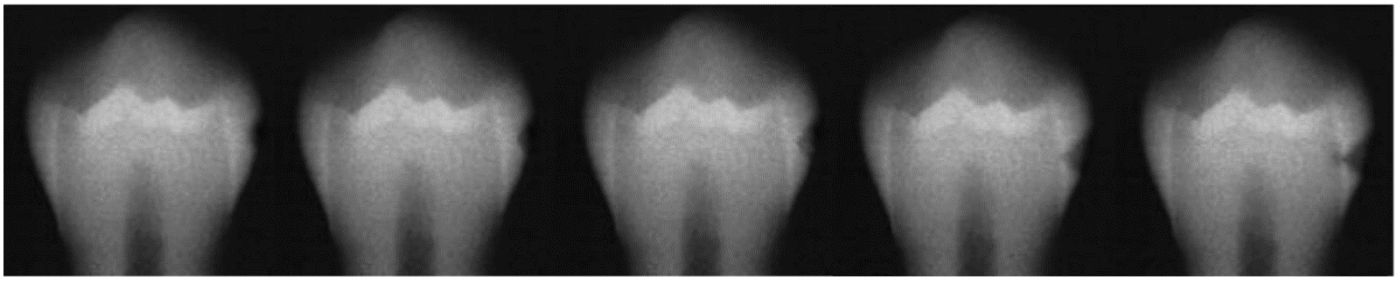

9. A 27-year-old male patient presents to your clinic. He has not gone to a dentist in five years and requests an exam and completion of any necessary treatment. You find he has good oral hygiene, a slight catch on the occlusal amalgam of 37, a sticky pit on the occlusal of 36 and dark stains along the margins of an amalgam on 35. How would you proceed?

- ☐ Restore 37, monitor 36, 35.
- ☐ Restore 36, 37, monitor 35.
- ☐ Restore 37, 36, 35.
- ☐ Restore 36, monitor 35, 37.

10. A 35-year-old female patient presents to your clinic. She is new to your practice, having recently moved to the city. She has a non-contributory medical history and fair oral hygiene. She tells you that at her last dentist visit two years ago, the dentist performed a complete examination and radiographs. She asks you how often she should have a complete examination and radiographs? What is your response?

- ☐ I perform complete examinations every year and radiographs whenever indicated.
- ☐ I perform complete examinations every year and radiographs every two years.
- ☐ I will do a complete examination for your initial visit and will only take radiographs if needed.
- ☐ I will need to do a complete examination and radiographs every year.

Now some questions about your clinical practice:

11. On average, how many hours a week do you currently work in clinical practice?

- ☐ Less than 20 hours
- ☐ 20-35 hours
- ☐ 35-50 hours
- ☐ More than 50 hours

12. On a per chair basis, how much do you feel you have to bill **per hour** to be profitable?

- ☐ Less than $100
- ☐ $100 - 200
- ☐ $200 - 300
- ☐ $300 - 400
- ☐ $400 - 500
- ☐ $500 - 600
- ☐ $600 - 700
- ☐ $700 - 800
- ☐ $800 - 900
- ☐ $900 - 1000
- ☐ Greater than $1000

13. What percentage of your patients are covered by:
(Percentages should add up to 100%.)

Private insurance	%
Public insurance	%
Out-of-pocket	%
**Total**	**100%**

14. Considering your primary practice, how many dentists are in the practice, including yourself?

- [ ] 1
- [ ] 2-4
- [ ] 5 or more

15. Which describes your current employment status?
(Please check all that apply.)

- [ ] Sole owner
- [ ] Partner
- [ ] Associate

If you picked **Sole owner** or **Partner** in question 15, please answer the following questions.

If not, please go to **question 22**.

16. How many practices do you own/partner in? __________

17. What percentage of time do you spend in each of your practices?
(Percentages should add up to 100%.)

Primary practice you are an owner/partner	%
Secondary practice you are an owner/partner	%
Tertiary practice you are an owner/partner	%
Remaining practices you are an owner/partner	%
Practices you are an associate	%
**Total**	**100%**

18. Considering your current situation, do you feel your practice loans are:

- [ ] Small
- [ ] Medium
- [ ] Large
- [ ] I do not have outstanding loans

Please answer the following questions for your **primary practice**.

19. How old is your practice?

- [ ] 0 to 5 years
- [ ] 6 to 10 years
- [ ] > 10 years

20. How many hygienists do you employ?

- [ ] 0
- [ ] 1
- [ ] 2
- [ ] 3
- [ ] 4
- [ ] 5 or more

21. How many **total hours a week** does the hygienist work?
(If you have more than one hygienist, please total their hours.)

- [ ] Less than 20 hours
- [ ] 20-35 hours
- [ ] 35-50 hours
- [ ] More than 50 hours

Now more case scenarios. Please select one answer for each scenario.

22. A 25-year-old female reporter on a local TV channel presents to your clinic for her regular check-up. She has a non-contributory medical history and good oral hygiene. She has discoloured 11 and 21 facial composite restorations and extrinsic stains on her maxillary anterior teeth. She states, "I hate those stains, they make me look ugly." How would you proceed?

- [ ] Recommend in-office whitening and then replace the old composites with new fillings.
- [ ] Recommend full-coverage porcelain crowns on the anterior teeth.
- [ ] Recommend porcelain veneers on the maxillary anteriors.

☐ Recommend porcelain veneers on maxillary and mandibular anteriors.

23. A 10-year-old patient presents to your clinic for a check-up. He has no medical conditions and good oral hygiene. The exam reveals sticky pits on 54 O, 64 O, 36 O and 46 O. What treatment would you recommend?

☐ Place sealants on 54, 64, 36, 46.
☐ Monitor 54, 64, and place sealants on 36, 46.
☐ Place restorations on 54, 64, 36, 46.
☐ Place sealants on 54, 64, and restorations on 36, 46.

24. A 48-year-old male present to your clinic. He hasn't been to a dentist in six years, and his chief complaint is having sensitivity when biting on 36. He has a non-contributory medical history and good oral hygiene. You find that teeth 15, 26 and 36 are chipped. In 15 and 26, this involves only the enamel, and on 36, this involves the dentine. The patient mentions he may have a clenching habit. What do you do next?

☐ Restore 15, 26, 36, and fabricate a night guard.
☐ Restore 15, 26, place a crown on 36, and fabricate a night guard.
☐ Monitor 15, 26, restore 36, and fabricate a night guard.
☐ Place crowns on 15, 26, 36 and fabricate a night guard.

Now some questions about your student loans:

25. Did you have any student loans?

☐ Yes
☐ No

<u>If you answered No</u>, please go to question 28.

26. How long did it take to pay off your student loans?

- ☐ Less than 1 year
- ☐ 1 to 5 years
- ☐ 5-10 years
- ☐ More than 10 years
- ☐ My student loans are not yet paid off

27. Do you feel your student loans are/were:

- ☐ Small
- ☐ Medium
- ☐ Large

Please read the following case scenarios and select one answer for each.

28. A 50-year-old male patient comes to your clinic for a check-up. The patient has a non-contributory medical history. His radiographs reveal a periapical radiolucency on the 46, which is an abutment for a 3-unit bridge from 44 to 46. The patient reports no pain and never having a problem concerning this site. How would you proceed?

- ☐ Remove the bridge, perform endodontic treatment on 46, and place a new bridge.
- ☐ Monitor the periapical lesion and advise the patient to come back for recalls.
- ☐ Remove the bridge, perform endodontic treatment on 46, place crowns on 46, 44, and a 45 implant.
- ☐ Perform endodontic treatment on 46 through the crown.

29. A 65-year-old female patient has 2mm of gingival recession on teeth 34 and 35, with moderate abrasion on teeth 24 and 25. She also has stained and sticky surfaces on the exposed buccal roots of 34 and 35. She takes medications that contribute to mild xerostomia. She demonstrates good manual dexterity and good oral hygiene. What would you recommend for this patient?

- ☐ Place her on a 3-month recall and apply fluoride varnish at every visit.

- [ ] Restore 34, 35, place her on a 3-month recall and apply fluoride varnish at every visit.
- [ ] Restore 24, 25, 34, 35, place her on a 3-month recall and apply fluoride varnish at every visit.
- [ ] Restore 34, 35, place her on a 6-month recall and refer her to a periodontist for gingival grafts on the exposed root surfaces.

30. An 8-year-old female patient presents to your clinic. Upon clinical examination, you find she has a normal tooth eruption pattern, excellent oral hygiene and no caries. What is your recommended treatment plan and recall frequency for this patient?

- [ ] Do a complete exam, prophy, give fluoride, and advise a 9-month recall.
- [ ] Do a complete exam, take radiographs, perform scaling, give fluoride, and advise a 9-month recall.
- [ ] Do a complete exam, take radiographs, perform scaling, prophy, give fluoride, and advise a 6-month recall.
- [ ] Do a complete exam, perform scaling, give fluoride, and advise a 6-month recall.

Now some questions about your clinical work.

31. Not including hygiene checks, how many patients do you see on an average day?

- [ ] Less than 5
- [ ] 5 - 7
- [ ] 7 - 9
- [ ] 9 - 11
- [ ] 11 - 13
- [ ] More than 13

32. On an average day, what would you estimate your **personal** gross billings to be?

- [ ] Less than $1000
- [ ] $1000 - 1500
- [ ] $1500 - 2000
- [ ] $2000 - 2500
- [ ] $2500 - 3000
- [ ] $3000 - 3500
- [ ] $5500 - 6000
- [ ] $6000 - 6500
- [ ] $6500 - 7000
- [ ] $7000 - 7500
- [ ] $7500 - 8000
- [ ] $8000 - 8500

☐	$3500 - 4000	☐	$8500 - 9000
☐	$4000 - 4500	☐	$9000 - 9500
☐	$4500 - 5000	☐	$9500 - 10,000
☐	$5000 - 5500	☐	More than $10,000

33. Are you satisfied with the level of busyness in your practice?

- ☐ Very satisfied
- ☐ Somewhat satisfied
- ☐ Not sure
- ☐ Somewhat dissatisfied
- ☐ Very dissatisfied

34. During a typical work week, what percentage of **your** time is devoted to each of the following procedures? **(Percentages should add up to 100%.)**

Diagnostic and preventive (exam, x-rays, scaling, prophylaxis, sealants, fluoride, etc.)	%
Restorative (fillings)	%
Crowns (tooth-supported and implant-supported)	%
Extractions	%
Implant surgery	%
Orthodontics (including Invisalign)	%
Cosmetic Dentistry	%
Full-mouth reconstruction	%
Other: ______________________________	%
**Total**	**100%**

35. What type of clinical technologies do you use in your practice? **(Please check all that apply.)**

- ☐ Cone Beam Computed Tomography (CBCT)
- ☐ Panoramic radiographs
- ☐ Cephalometric radiographs
- ☐ Fluorescence visualization devices (e.g. Velscope)
- ☐ Cerec machine (CAD/CAM)
- ☐ Laser periodontal debridement devices (e.g. Periowave)
- ☐ Caries detection devices (e.g. VistaProof, Canary)

☐ Other: ____________________________

36. How often do you refer patients to other practitioners for the categories listed below? **(Check one response for each item.)**

	Never	Occasionally	Often	Always
Periodontics				
Complex periodontics				
Prosthodontics - fixed				
Prosthodontics - removable				
Endodontics				
Complex endodontics				
Extractions				
Complex extractions				
Orthodontics				
Medically compromised				
Behaviour management				

37. For the past two years, **please check the <u>three subject areas</u>** where you took the most continuing education:

☐ Practice management
☐ Oral medicine/pathology
☐ Infection control
☐ Radiology
☐ Medical emergencies
☐ Periodontics
☐ Orthodontics
☐ Restorative/cosmetic dentistry
☐ Endodontics
☐ Removable prosthodontics

- [ ] Treatment planning
- [ ] Paediatric dentistry
- [ ] Malpractice
- [ ] TMD
- [ ] Implants
- [ ] Anaesthesia
- [ ] Fixed prosthodontics
- [ ] Oral surgery
- [ ] Other: ________________________

38. Dentists have various roles, such as being a health care professional and a business person. In terms of the relative balance between the two roles, please place an 'X' on the line below to describe where you perceive yourself to be.

|——————————|——————————|

Health care Professional — Business Person

39. Do you perceive other dentists as colleagues or competitors? Please place an 'X' on the line below to describe your relationship.

|——————————|——————————|

Colleague — Competitor

40. How would you rate the amount of pressure you feel from competition from other dental practices?

- [ ] Small amount
- [ ] Medium amount
- [ ] Large amount
- [ ] I do not feel pressure from other dental practices.

Now we would like to hear your perspective on some issues regarding the profession and the dentist-patient relationship:

41. To which patient populations do you think you have a duty to care? Please state your level of agreement for each of the following groups.

Group	Strongly Disagree	Disagree	Not Sure	Agree	Strongly Agree

Population at large					
All patients in my practice					
Low-income children					
Low-income adults					
Low-income seniors					
Adults on social assistance					

42. Please make one selection for each statement.

	**Strongly Disagree**	**Disagree**	**Not Sure**	**Agree**	**Strongly Agree**
It is important to understand a patient's culture and background in order to treat a patient's illness.					
Patients should be treated as if they were partners with the dentist, equal in power and status.					
That I provide an equally good standard of care whether working on publicly or privately insured patients is important to me.					
Reducing inequalities in oral health across the population is important to me.					
Dentists should lobby for dental benefits for the disadvantaged.					

Now some statements about your dental practice.

43. Please make one selection for each statement.

	**Strongly Disagree**	**Disagree**	**Not Sure**	**Agree**	**Strongly Agree**
Identifying new business opportunities for the practice is important to me.					
Positioning the practice in the marketplace is important to me.					
Having remuneration in line with my years of training/skills is important to me.					
Thinking about the financial implications for the practice when I					

advise patients of treatment options is important to me.					

Finally, some questions about you and your family:

44. Are you the primary income earner in your household?

- ☐ Yes
- ☐ No
- ☐ Me and my partner contribute equally
- ☐ Prefer not to say

45. How many dependents do you have (people you support financially)?

- ☐ 0
- ☐ 1
- ☐ 2-4
- ☐ 5 or more

46. Approximately, what is your **personal** after tax income?

- ☐ Less than $100,000/year
- ☐ $100,000 – 150,000/year
- ☐ $150,000 – 200,000/year
- ☐ $200,000 – 250,000/year
- ☐ $250,000 – 300,000/year
- ☐ $300,000 – 350,000/year
- ☐ $350,000 – 400,000/year
- ☐ $400,000 – 450,000/year
- ☐ $450,000 – 500,000/year
- ☐ $500,000 – 550,000/year
- ☐ $550,000 – 600,000/year
- ☐ $600,000 – 650,000/year
- ☐ $650,000 – 700,000/year
- ☐ $700,000 – 750,000/year
- ☐ More than $750,000
- ☐ Prefer not to say

If you have any other comments you would like to add, please use the space below.

____
____
____
____
____
____
____
