# Supplementary material for: What influences the clinical decision-making of dentists? A cross-sectional study
Source: PLoS One. 2020 Jun 5;15(6):e0233652. doi: 10.1371/journal.pone.0233652 (PMC7274387; doi:10.1371/journal.pone.0233652)
Supplement: S2 Appendix — (PDF) [file pone.0233652.s002.pdf]

Perception of student loans	Perceived professional role	Perception of other dentists	Perceived pressure from other dental clinics	Primary income earner	Number of dependents	Annual after-tax income
-0.472	0.079	0.005	-0.132	0.181	-0.221	0.001
0.238	-0.054	-0.009	0.038	-0.404	-0.049	-0.195
0.286	0.058	-0.032	-0.026	-0.123	0.031	-0.163
0.491	-0.073	0.011	0.142	-0.162	0.193	0.012
-0.337	0.046	-0.015	-0.097	0.022	0.089	0.070
-0.368	0.021	-0.124	-0.157	0.036	-0.060	-0.015
0.011	0.050	0.001	0.027	-0.003	0.008	0.055
0.073	0.006	-0.018	-0.038	-0.007	0.022	-0.037
-0.133	-0.092	0.025	0.009	0.015	-0.046	-0.039
0.138	0.028	0.059	0.083	0.130	0.138	0.223
0.055	0.096	0.054	0.050	0.055	0.059	0.269
0.074	0.021	-0.064	-0.071	-0.060	-0.018	0.048
-0.023	0.078	0.069	0.104	0.092	0.213	0.212
-0.290	0.015	-0.033	-0.102	0.134	0.025	0.164
0.003	0.064	-0.014	-0.050	0.063	0.123	0.297
0.008	0.049	-0.100	-0.073	0.068	0.146	0.312
-0.015	-0.027	0.015	0.075	-0.116	-0.081	-0.228
0.111	-0.077	0.009	-0.026	0.043	0.021	0.030
-0.112	0.114	-0.025	-0.043	0.063	0.054	0.186
0.397	-0.009	0.060	0.065	0.005	0.100	0.015
-0.043	-0.017	0.002	-0.053	0.097	0.027	0.342
-0.026	0.018	0.002	-0.040	0.121	0.165	0.511
0.033	0.006	0.215	0.425	0.004	0.019	-0.227
-	-0.019	0.050	0.090	-0.069	0.039	-0.133
	-	0.125	0.024	0.030	-0.014	0.071
		-	0.407	0.090	0.013	0.003
			-	0.031	0.078	-0.037
				-	0.040	0.168
					-	0.180
						-

Percentage of treatment services	Percentage of elective services	Time to pay off student loans	Number of patients seen/day	Personal gross billing	Satisfaction with practice busyness
-0.146	0.105	-0.210	0.006	-0.153	0.028
0.021	-0.095	0.100	-0.117	-0.121	-0.008
-0.042	-0.011	0.214	-0.105	-0.130	0.008
0.150	-0.089	0.176	0.007	0.174	-0.040
-0.116	0.077	0.015	0.037	-0.066	0.017
-0.060	0.064	-0.125	0.147	-0.064	-0.098
0.048	0.008	0.086	-0.014	0.049	0.044
0.035	-0.094	-0.014	0.013	-0.021	-0.035
-0.132	0.123	-0.0130	0.004	-0.053	-0.023
-0.046	0.083	0.100	0.174	0.216	-0.026
-0.066	0.257	0.010	0.184	0.498	0.034
0.056	0.023	-0.065	0.062	0.076	-0.141
-0.109	0.091	0.093	0.075	0.136	0.095
0.002	0.027	-0.146	0.090	0.002	-0.048
0.259	0.119	0.015	0.163	0.379	-0.214
0.165	0.097	0.036	0.232	0.384	-0.284
-0.562	-0.332	-0.038	-0.107	-0.301	0.172
-	-0.593	-0.006	0.056	0.003	-0.081
	-	0.041	0.040	0.286	-0.073
		-	0.019	-0.039	-0.038
			-	0.413	-0.179
				-	-0.202
					-

Amount billed to be profitable	Number of dentists	Practice ownership	Practice age	Number of hygienists	Number of hygiene hours	Percentage of diagnostic and preventive services
-0.072	-0.078	-0.066	0.364	-0.182	-0.179	0.063
-0.033	0.096	-0.084	-0.231	0.003	-0.043	0.073
-0.015	-0.007	0.060	-0.147	-0.133	-0.235	0.060
0.069	0.066	0.59	-0.331	0.174	0.203	-0.086
-0.013	-0.065	0.139	0.257	-0.049	-0.056	0.057
-0.142	-0.071	0.090	0.475	0.093	-0.004	0.011
0.053	0.071	0.010	0.004	0.194	0.105	-0.064
-0.009	-0.027	0.003	0.001	-0.100	-0.076	0.056
-0.077	-0.081	-0.021	-0.009	-0.191	-0.071	0.028
0.113	-0.170	0.384	-0.105	0.031	0.074	-0.031
-	0.079	0.012	-0.063	0.172	0.144	-0.190
	-	-0.380	0.050	0.314	0.324	-0.088
		-	-	-	-	0.035
			-	0.089	0.103	-0.030
				-	0.548	-0.423
					-	-0.334
						-

Years of practice	Years of practice in Canada	Percentage of private insurance patients	Percentage of public insurance patients	Percentage of out of pocket patients	Number of hours worked/week
0.517	0.481	-0.81	-0.004	0.144	-0.227
-0.187	-0.094	-0.002	0.072	-0.102	-0.030
0.065	-0.127	-0.121	0.171	-0.045	0.059
-0.560	-0.460	0.086	-0.011	-0.129	0.243
-	0.487	-0.037	0.004	0.056	-0.130
	-	-0.036	-0.027	0.115	-0.067
		-	-0.810	-0.514	-0.002
			-	-0.087	0.022
				-	-0.029
					-

Appendix 2. The correlation matrix of the independent variables

	Age	Gender	Place of initial training	Year of graduation
Age	-	-0.355	-0.055	-0.906
Gender		-	0.265	0.319
Place of initial training			-	-0.009
Year of graduation				-
Years of practice				
Years of practice in Canada				
Percentage of public insurance patients				
Percentage of private insurance patients				
Percentage of out of pocket patients				
Number of hours worked/week				
Amount billed to be profitable				
Number of dentists				
Practice ownership				
Practice age				
Number of hygienists				
Number of hygiene hours				
Percentage of diagnostic and preventive				
Percentage of treatment services				
Percentage of elective services				
Time to pay off student loans				
Number of patients seen/day				
Personal gross billing				
Satisfaction with practice busyness				
Perception of student loan				
Perceived professional role				
Perception of other dentists				
Perceived pressure from other dentists				
Primary income earner				
Number of dependents				
Annual after-tax income				
