# Supplementary material for: What influences the clinical decision-making of dentists? A cross-sectional study
Source: PLoS One. 2020 Jun 5;15(6):e0233652. doi: 10.1371/journal.pone.0233652 (PMC7274387; doi:10.1371/journal.pone.0233652)
Supplement: S3 Appendix — (PDF) [file pone.0233652.s003.pdf]

Appendix 3. Demographic comparison

	Survey (%)	Ontario Dental Association (%)	Canadian Dental Association (%)
**Gender**			
Male	65.5	62.0	61.4
Female	34.5	38.0	35.6
**Age**			
40 and under	14.4	29.0	28.4
41 – 50	25.7	25.0	24.3
51 – 60	30.4	22.0	22.4
61 and older	29.5	19.0	20.3
**Year of graduation**			
Before 1970	3.4	3.0	2.9
1970 - 1979	17.8	11.0	12.0
1980 - 1989	29.3	20.0	21.4
1990 - 1999	28.7	22.0	23.6
2000 - 2009	15.5	24.0	25.2
2010 - 2016	5.3	20.0	12.9
**Place of initial dental training**			
Canadian dental school	75.4	71.0	76.9
American dental school	7.9	12.0	8.4
International dental school	16.7	17.0	12.7
**Practice ownership**			
Owner/Partner	73.3	66.0	N/A
Associate	26.7	34.0	N/A
